# The role of 14‐3‐3 proteins in cell signalling pathways and virus infection

**DOI:** 10.1111/jcmm.16490

**Published:** 2021-04-01

**Authors:** Jiaqi Liu, Shengliang Cao, Guofei Ding, Bin Wang, Yingchao Li, Yuzhong Zhao, Qingyuan Shao, Jian Feng, Sidang Liu, Liting Qin, Yihong Xiao

**Affiliations:** ^1^ Department of Fundamental Veterinary Medicine College of Animal Science and Veterinary Medicine Shandong Agricultural University Tai'an China; ^2^ Shandong Provincial Key Laboratory of Animal Biotechnology and Disease Control and Prevention Shandong Agricultural University Tai’an China; ^3^ Shandong New Hope Liuhe Group Co., Ltd. Qingdao China; ^4^ Qingdao Jiazhi Biotechnology Co., Ltd. Qingdao China

**Keywords:** 14‐3‐3 proteins, biological function, innate immunity, viral infection

## Abstract

14‐3‐3 proteins are highly conserved in species ranging from yeast to mammals and regulate numerous signalling pathways via direct interactions with proteins carrying phosphorylated 14‐3‐3–binding motifs. Recent studies have shown that 14‐3‐3 proteins can also play a role in viral infections. This review summarizes the biological functions of 14‐3‐3 proteins in protein trafficking, cell‐cycle control, apoptosis, autophagy and other cell signal transduction pathways, as well as the associated mechanisms. Recent findings regarding the role of 14‐3‐3 proteins in viral infection and innate immunity are also reviewed.

## 1 INTRODUCTION

14‐3‐3 proteins are widely distributed in various organs and tissues of both plants and animals, and play roles in pleiotropic functions, including cell biology and cell signalling. There are seven 14‐3‐3 subtypes (α/β, γ, ε, η, σ, τ [also called θ] and ζ/δ) in mammals.^1^ The molecular weight of 14‐3‐3 protein is approximately 30 kD and PI is 4‐5.^2^ The N‐terminal and C‐terminal of the 14‐3‐3 proteins represent key functional domains. The N‐terminal affects the binding of 14‐3‐3 proteins to different membranes, whereas the C‐terminal is directly involved in the protein–protein interaction.^2^

## 2 LIGANDS OF 14‐3‐3 PROTEINS

Several hundred partners have been identified to bind 14‐3‐3 proteins.^3^ It has been reported that the 14‐3‐3 recognition sequences are diverse. Many ligands contain conserved phosphorylated serine (Ser)/threonine (Thr) sequence motifs.^2^ All isoforms recognize two high‐affinity phosphorylation‐dependent 14‐3‐3 binding motifs: RSXpSXP (mode I) and RXUXpSXP (mode II) (U as an aromatic or aliphatic amino acid, X as any amino acid).^4^ Besides, the characteristic binding of protein C‐termini and 14‐3‐3 was proposed as mode III.^4^ There are some predictive value of 14‐3‐3 consensus motifs; however, the motifs are poorly predicted simply based on sequences.^2^

The Ser‐rich motif is also a 14‐3‐3–binding motif and includes RX_1‐2_SX_2‐3_S (where X represents a basic amino acid), of which at least one serine must be phosphorylated.^2^ For example, Cbl (RHpS^619^LPFpS^623^, RLGpS^639^TFpS^642^) and PKCμ (RLpS^205^NVS^208^, RTSpS^219^AELpS^223^) contain this type of sequence motif.^2^ There are also non‐acidified motifs in the mitochondrial directional sequences.^2^

14‐3‐3–binding motifs are also found in viral proteins. The NS3 protein of dengue virus directly binds to 14‐3‐3ε protein through its highly conserved phosphomimetic RxEP motif. The NS3 protein of Zika virus contains a cellular 14‐3‐3–binding motif that can directly interact with 14‐3‐3ε and 14‐3‐3η.^5^ There are still several unknown motifs in viral proteins. For example, BPLF1 of Epstein‐Barr virus (EBV), UL48 of human cytomegalovirus (HCMV) and ORF64 of Kaposi's sarcoma‐associated herpesvirus (KSHV) are homologues that can interact with 14‐3‐3, but there is no information available on the binding site(s) involved.^6^, ^7^ It was reported that the phosphorylated Ser31 on Hepatitis B virus (HBV) protein X (HBx) constituted a RPLphosphoS31 GP (R, arginine; P, proline; L, leucine; S, serine; G, glycine) motif for 14‐3‐3ζ docking.^8^ The combined activity of human papillomavirus (HPV) E6 and E7 oncoproteins can cause cervical cancer. There is a PDZ binding motif (PBM) at the extreme carboxyl end of E6 oncoprotein, which is important for the interaction between E6 and 14‐3‐3ζ.^9^ Moreover, 14‐3‐3 proteins also interact with RNA and promote the replication of porcine circovirus type 2 via enhancement of autophagy by targeting microRNA‐30a‐5p.^10^

## 3 BIOLOGICAL FUNCTIONS OF 14‐3‐3 PROTEINS

By binding with hundreds of ligands, 14‐3‐3 proteins perform a multitude of regulatory functions, including molecular interactions, subcellular localization, scaffolding and stability. As a result, 14‐3‐3 proteins can participate in multiple cellular biological functions, including the cell cycle, apoptosis, autophagy, cell signal transduction and other cellular activities.

### 3.1 14‐3‐3 proteins in the cell cycle

**FIGURE 1:**
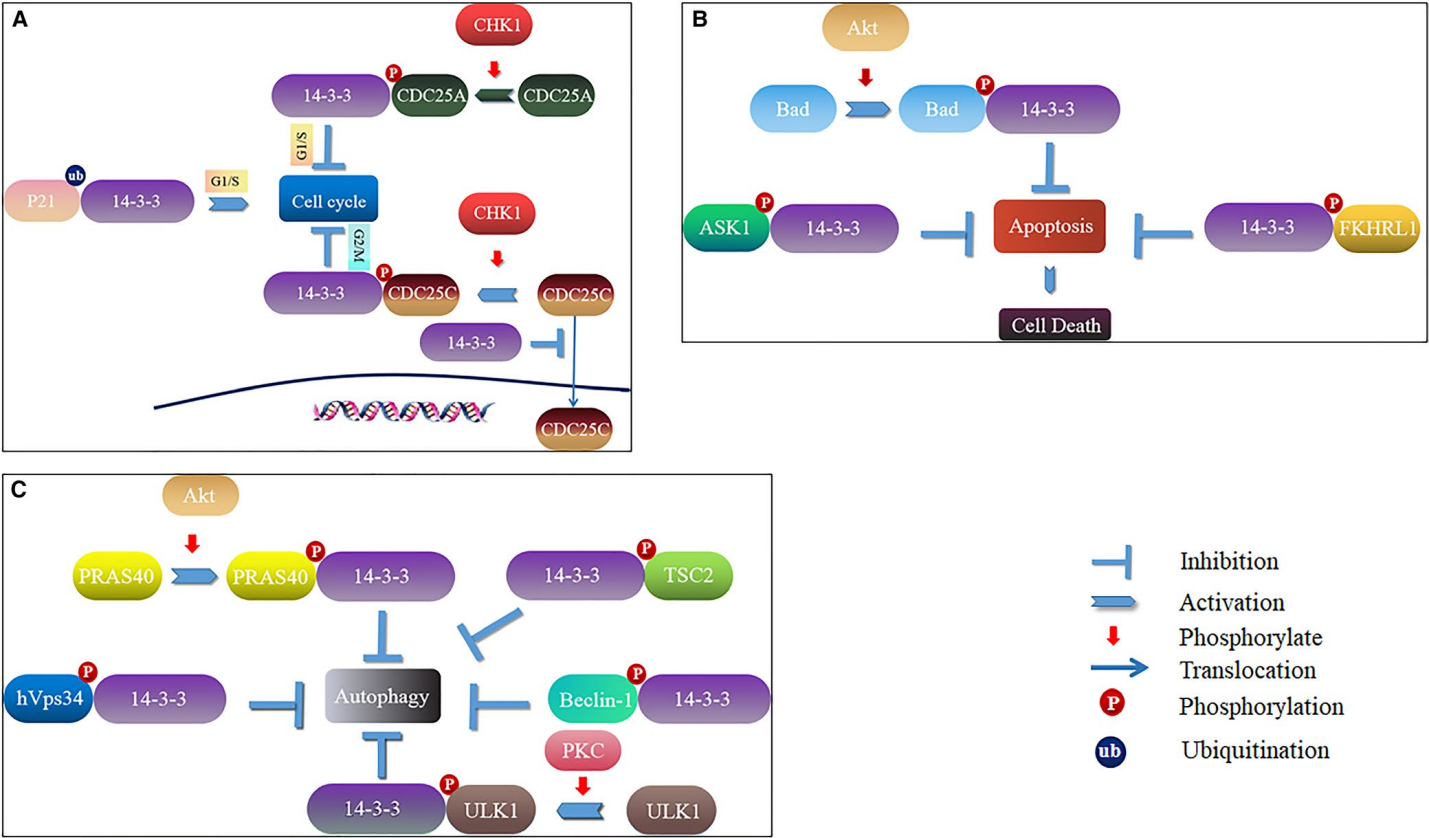
The main roles of 14‐3‐3 Proteins in cell signalling pathway. A, Regulation of 14‐3‐3 proteins in cell cycle. In G2/M phase, 14‐3‐3 proteins interact with CDC25C, which prevents CDC25C‐mediated interaction between CDK1 and Cyclin, thus blocking the cell cycle in M phase. CDC25C mediated by CHK1 is phosphorylated at Ser216 and binds to 14‐3‐3 proteins, making CDC25C blocked in the cytoplasm and inhibiting the cell cycle. In the G1/S phase, CHK1 kinase phosphorylates CDC25A at Ser178 and Thr507. Then, CDC25A binds to 14‐3‐3 proteins, thereby blocking the activation of the CDK/Cyclin complex, resulting in G1/S phase blockade. 14‐3‐3 proteins can degrade P21 in an ubiquitin‐dependent manner, thereby promoting the G1/S phase of the cell cycle. B, Regulation of 14‐3‐3 proteins in apoptosis. The AKT phosphorylates Ser136 on Bad, and then Bad interacts with 14‐3‐3 proteins, blocking BAD into the cytoplasm and not entering the mitochondria, thereby inhibiting apoptosis. The interaction of ASK1 with 14‐3‐3 proteins after the phosphorylation at Ser996, they form a complex, thereby inhibiting the activity of ASK1 kinase, thereby inhibiting apoptosis. As an anti‐apoptotic factor, 14‐3‐3 proteins interact with FKHRL1, which is phosphorylated at Thr24, to inhibit FKHRL1‐induced apoptosis. C, Regulation of 14‐3‐3 proteins in autophagy. 14‐3‐3 proteins can interact with phosphorylated PRAS40 which is at Ser183 and Ser221 and phosphorylated TSC2 at Ser939, thereby inhibiting the initiation of autophagy. 14‐3‐3 proteins interact with phosphorylated ULK1 at Ser555, blocking the formation of pre‐autophagosomes induced by ULK1, thereby inhibiting cellular autophagy. 14‐3‐3 proteins interact with hVps34 at a phosphorylated site Ser212, and the interaction between them hinders the activity of hVps34. Beclin‐1 is also an important molecule in the formation of autophagosomes. Beclin‐1 interacts with 14‐3‐3 proteins through phosphorylation at Ser234 and Ser295, thereby promoting tumourigenesis by inhibiting autophagy

The role of 14‐3‐3 proteins in the cell cycle was first discovered in yeast in the context of DNA damage repair.^11^ Ligands of 14‐3‐3 proteins include cell division cycle 25 phosphatases (CDC25s), cycle checkpoint kinase 1 (CHK1) and tyrosine kinase Wee1.^12^, ^13^, ^14^ The 14‐3‐3 proteins bind to the cell division cycle 25A (CDC25A) at Ser178 and Thr507, which are phosphorylated by CHK1 to block the interaction between CDC25A and cyclin‐dependent kinase 2 (CDK2), and then retard entry of cells into S phase (Figure 1A).^15^ p21 is an inhibitor of CDKs and a target gene of the transcription factor p53, which can regulate p53‐dependent G1 arrest and senescence.^16^ p21 is degraded by ubiquitin‐dependent and ubiquitin‐independent mechanisms, and 14‐3‐3 proteins play an important role in both ubiquitin‐dependent and ubiquitin‐independent p21 proteasome degradation (Figure 1A).

In mammalian cells, activation of the CDC2 protein kinase via dephosphorylation of CDC25C is a necessary step for cell entry into M phase.^17^ 14‐3‐3 proteins bind to CDC25C at Ser216, which is phosphorylated by CHK1.^18^ Binding of CDC25 to 14‐3‐3 proteins inhibits the activity of CDC25 phosphatase, thereby preventing cells from entering mitosis and inhibiting the cell‐cycle process (Figure 1A).^19^

The 14‐3‐3 proteins can also regulate transcription factors in cell cycle. FOXO is a transcription factor belonging to the forkhead family with characteristic pterygoid spiral DNA‐binding domains.^20^ The phosphoinositide 3‐kinase (PI3K)/protein kinase B (PKB, also known as Akt) signalling pathway is the main upstream pathway of FOXO.^21^ Binding of FOXO to 14‐3‐3 results in loss of its transcriptional activity and its role in regulating the cell cycle.^21^

### 3.2 14‐3‐3 proteins in apoptosis and autophagy

Bad is a proapoptotic member of the Bcl‐2 family. It was reported that phosphorylation of BAD at Ser111, Ser112 and Ser136 are related to the BAD/14‐3‐3 binding.^22^ The AKT phosphorylate Ser136 on Bad, and then Bad interacts with 14‐3‐3, blocking Bad into the cytoplasm and not entering the mitochondria, thereby inhibiting apoptosis.^23^ Apoptosis signal‐regulating kinase 1 (ASK1) interacts with 14‐3‐3 at Ser996 form a complex that inhibits the activity of ASK1 kinase and thus induces apoptosis.^24^ FKHRL1 (also known as FOXO3) is a target protein downstream of PI3K‐AKT that can promote apoptosis. The 14‐3‐3 proteins recognize the phosphorylated site Thr24 on FKHRL1. During this process, 14‐3‐3 acts as an anti‐apoptotic factor and inhibits FKHRL1‐induced apoptosis (Figure 1B).^25^ Besides, it was reported that Ser22 phosphorylation in FOXO1 prevented the binding of 14‐3‐3 proteins, which related to conformational changes within the NTD of FOXO1, steric and electrostatic effects.^26^

Macroautophagy, also called autophagy, is the most well‐studied type of autophagy. The first step of autophagy is autophagosome generation, followed by expansion of the membrane to form the phagophore, which is the primary double‐membrane sequestering compartment.^27^ 14‐3‐3 proteins play an important role in the initial formation by regulating unc51‐like autophagy activating kinase 1 (ULK1).^28^ Partner proline‐rich AKT substrate 40 (PRAS40) is a component of the mTORC1 complex. 14‐3‐3 proteins can interact with phosphorylated PRAS40 at Ser183 and Ser221, thereby inhibiting the initiation of autophagy.^29^ 14‐3‐3 proteins interact with phosphorylated ULK1 at Ser555, blocking the formation of pre‐autophagosomes induced by ULK1, thereby inhibiting cellular autophagy (Figure 1C).^29^, ^30^

PI3K/AKT/mTOR is a key pathway for the regulation of autophagy and comprises PI3K, AKT and mammalian target of rapamycin (mTOR).^31^ 14‐3‐3 proteins modulate the recruitment and activation of PI3K by directly interacting with insulin receptor substrate 1 (IRS‐1).^32^ Tuberous sclerosis 2 (TSC2) is phosphorylated at a conserved serine site, which leads to initiation of autophagy.^33^ Binding of 14‐3‐3 proteins to phosphorylated TSC2 at Ser939 can inhibit the initiation of autophagy, as well as protein synthesis and cell growth.^29^ 14‐3‐3 proteins may also control the autophagy process at a later stage by interacting with and regulating proteins involved in autophagosome formation (eg human‐derived version of yeast Vps34 [hVps34] and class III phosphoinositide 3‐kinase [PI3KC3]).^29^ Under normal growth conditions, the interaction between 14‐3‐3 and hVps34 at a phosphorylated site Ser212 hinders hVps34 activity. Beclin‐1 is also an essential molecule for autophagosome formation. Beclin‐1 interacts with 14‐3‐3 proteins via Ser234 and Ser295 phosphorylation, thereby promoting tumourigenesis by inhibiting autophagy (Figure 1C).^34^

## 4 FUNCTIONS OF 14‐3‐3 PROTEINS

Apart from biological functions of 14‐3‐3 proteins, they can participate in other multiple molecular interactions. 14‐3‐3 proteins are involved in functions of subcellular localization, scaffolding and stability.

### 4.1 14‐3‐3 proteins alter the nuclear trafficking of ligands

14‐3‐3 proteins have an effect on nuclear–cytoplasm protein shuttling. The nuclear export signal is highly conserved in 14‐3‐3 sequences. Binding with 14‐3‐3 proteins will hide the nuclear localization sequence (NLS) in the ligands and then regulate their functions.^35^

Caspase‐2 is an apical protease responsible for proteolysis of the cell substrate and is directly involved in the apoptosis signalling cascade. It is the only known caspase that shuttles through the nucleus.^36^ Caspase‐2 interacts with 14‐3‐3 in a phosphorylation‐dependent manner that obscures the NLS and blocks its nuclear trafficking.^37^ Structural analysis confirmed that phosphorylated caspase‐2 and 14‐3‐3ζ form a compact and rigid complex to prevent caspase‐2 activation.^38^ Grb2‐related regulatory factor of ERK/MAPK1 (GAREM1) is an adaptor protein involved in the epidermal growth factor (EGF) pathway. Nuclear localization of GAREM1 depends on the NLS, which is located in the N‐terminal cysteine‐containing all‐in‐Themis (CABIT) domain. Binding of 14‐3‐3ε to GAREM1 masks the NLS in its CABIT domain.^39^

It is believed that 14‐3‐3 proteins participate in regulation of the subcellular localization of the FOXO forkhead transcription factor. 14‐3‐3 proteins can competitively bind to FOXO and block binding of the target DNA, and thus interfere with the NLS functionality.^40^ Specifically, 14‐3‐3ζ functions as a molecular hood that covers the DNA‐binding interface of FOXO4 and blocks its interaction with the target DNA.^41^

14‐3‐3 proteins can also indirectly influence nuclear–cytoplasm protein shuttling. severe acute respiratory syndrome (SARS) outbroke in 2002/2003, which caused by severe acute respiratory syndrome coronavirus (SARS‐CoV).^42^ The binding of 14‐3‐3 with nucleocapsid (N) resulted in the translocation of phosphorylated N protein of SARS‐CoV from the nucleus to the cytoplasm. N protein could downregulate the expression of 14‐3‐3θ, leading to the accumulation of phosphorylated N protein in the nucleus.^43^ The raging coronavirus disease 2019 (COVID‐19) caused by the SARS‐CoV‐2 virus has brought a global crisis with its deadly spread all over the world.^44^, ^45^ Similar to SARS‐CoV, nucleocapsid (N) protein of SARS‐CoV‐2, which has nucleocytoplasmic shuttle function, is involved in viral genome packaging and contains several phosphorylation sites. N protein can bind to 14‐3‐3 at phosphorylated site, among which Ser197 is the key site. The association of phosphorylated N protein and 14‐3‐3 could regulate nucleocytoplasmic shuttling and other functions of N, and could also hijack cellular pathways by 14‐3‐3 sequestration.^46^ 14‐3‐3 proteins also cause the relocation of other proteins. For example, after being phosphorylated, the Ser216 of CDC25 proteins can combine with 14‐3‐3ε to form a complex and block its binding to importin α and enter the nucleus.^47^ After phosphorylation of the Ser246, Ser467 and Ser632 sites on histone deacetylase 4 (HDAC4), 14‐3‐3 proteins can interact with it, leading to relocation of HDAC from the nucleus to the cytoplasm.^48^

### 4.2 Stabilization of protein structure and activity

The 14‐3‐3 protein subunits have two ligand‐binding sites that facilitate close proximity for the interaction between 14‐3‐3 and their ligands.^49^ 14‐3‐3 proteins play an essential role in regulating signal transduction by acting as “scaffolds” or “anchors” that stabilize the protein structure and kinase activity.

Raf‐1 protein kinase is the main activator of the ERK–MAPK pathway.^50^ 14‐3‐3 proteins function as critical cofactors in Raf‐1 activation: they induce and maintain a protein state that is competent for both ATP‐binding and mitogen‐activated protein kinase/extracellular signal‐regulated kinase (MEK) phosphorylation.^50^ The activated GTP‐binding protein Ras directly interacts with Raf‐1 and recruits it to the cytoplasmic membrane, where Raf‐1 is then activated. During this activation, 14‐3‐3 proteins play a dual role: (1) they maintain the Raf‐1 inactive state when no activation signal is available; and (2) they activate Raf‐1 in the presence of an activation signal and stabilize its active conformation.^2^

In brain synapses, the cytomatrix at the active zone (CAZ), a specific area of the presynaptic plasma membrane, limits the release of neurotransmitters and is closely related to signal transduction between synapses.^51^ Bassoon is a component protein of the CAZ that targets the correct presynaptic release site and regulates neurotransmitter release. S2845 phosphorylation of Bassoon provides a site for 14‐3‐3 binding, which then functions as a scaffold protein to regulate presynaptic signal transduction in the cytoplasm.^52^ 14‐3‐3 proteins serve as a scaffold for protein–protein interactions and can interact with MEKK1, MEKK2 and MEKK3.^53^ 3‐Phosphoinositide‐dependent protein kinase‐1 (PDK1) is an important protein kinase in the PI3K–AKT pathway that can phosphorylate ATK.^54^ Studies have shown that the Ser241 site of PDK1 phosphorylates and regulates 14‐3‐3 binding, which negatively regulates the PI3K–AKT pathway.^55^

In addition, 14‐3‐3 proteins are involved in the regulation of signal transduction in plant cells. Studies have shown that a number of signalling proteins can interact with 14‐3‐3 proteins in plants (eg calcium‐responsive protein kinase 1 (CPK1), calcium‐dependent protein kinase 2 (CDPK2), and wheat protein kinase 4 (WPK4)).^56^ Interaction between 14‐3‐3 proteins and CPK1 affects its activity and regulates the metabolism of carbon and nitrogen in plants.^57^ WPK4 is a protein kinase responsible for controlling the nitrogen metabolism pathway. Interaction between 14‐3‐3 and WPK4 depends on phosphorylation and thus regulates metabolite decomposition.^58^ Among the cell signal transduction pathways, phosphorylation and dephosphorylation are the most important events associated with signal transduction. 14‐3‐3 proteins play an essential role in this process because they are phosphorylated and can supplement the phosphorylation events required to complete the signal transduction cascade.^59^

### 4.3 Other functions of 14‐3‐3 proteins

The 14‐3‐3 proteins have a greater number of functions than was previously recognized. 14‐3‐3 proteins bind to target proteins via phosphorylation (eg to the adrenergic α2 receptor and glucocorticoid receptor), thereby regulating a large number of signal transduction pathways at different levels and causing transcriptional activation or inhibition of related genes.^60^, ^61^ It has been reported that 14‐3‐3σ can promote fibroblast migration and inhibit collagen production, and it, therefore, plays an important role in both tissue homeostasis and repair.^62^ 14‐3‐3 proteins also regulate cell signal transduction processes by interacting with transcription factors, such as TFIIB, TATA‐box‐binding protein 2 (TBP2/TRF3), VP1, Eosinophil granule major basic protein 1 (EmBP1), and repression of shoot growth (RSG).^63^

## 5 ROLE OF 14‐3‐3 PROTEINS IN VIRAL INFECTION AND INNATE IMMUNITY

The innate immune system is the first stage of defence against invaders, including viruses, bacteria, parasites and toxins, as well as sensing wounds or trauma. Activation of the innate immune response is induced by activation of pattern recognition receptors expressed by innate immune cells, which serve to identify PAMPs.^64^ The 14‐3‐3 proteins play a major role in pathogen recognition and intracellular signalling of innate immunity that initiates the immune response to DNA and RNA virus infection primarily by regulating the TLR and retinoic acid‐inducible gene I (RIG‐I)‐like receptor (RLR) signalling pathways.

### 5.1 14‐3‐3 proteins modulate antiviral defences via the TLR signalling pathway

**FIGURE 2:**
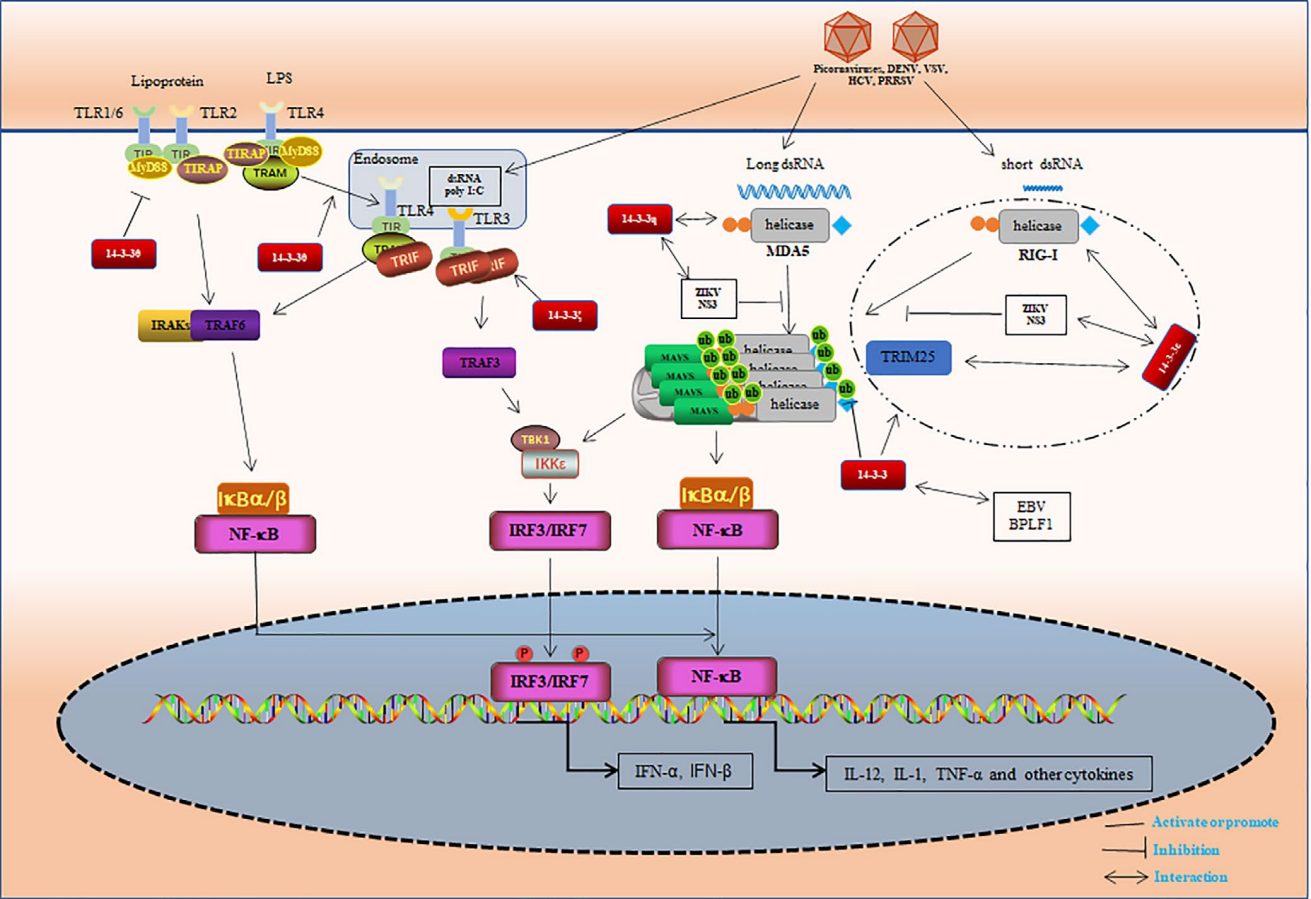
14‐3‐3 proteins regulate innate immunity. RIG‐I and MDA5 recognize both short dsRNA and long dsRNA viruses, respectively, and are then transferred to the mitochondria to interact with MAVS, induce IRF3, IRF7 and NF‐κB activation, promoting the production of IFN and various inflammatory factors. 14‐3‐3ε plays a crucial role in the transfer of RIG‐I to mitochondrial MAVS and is a partner of RIG‐I interaction and promotes the translocation complex containing RIG‐I, 14‐3‐3ε and TRIM25 formation (the dotted circle indicated in the figure). 14‐3‐3ε interacts with RIG‐I and TRIM25, and stabilizes the interaction between the RIG‐I and TRIM25 proteins. 14‐3‐3η promotes the transport of MAD5 from the cytoplasm to the mitochondrial membrane through interaction with MDA5, thereby enhancing and boosting the MAD5‐mediated antiviral response. However, the interaction between the ZIKV NS3 protein and 14‐3‐3ε hinders the transduction of RIG‐I, while interaction with 14‐3‐3η hinders the transduction of MDA5, further inhibiting innate immunity and promoting its own virus replication. In addition, the interaction of the EBV‐encoded homologue, BPLF1 and 14‐3‐3 proteins promotes the formation of the translocation complex and inhibits RIG‐I ubiquitination to block the innate immune response. Toll‐like receptors (TLRs) are activated after recognizing PAMPs, recruiting the proximal cytoplasmic Toll/IL‐1 receptor (TIR) domain‐containing adaptor proteins. Both lipoproteins and LPS are recognized on the cell surface by a heterodimer of TLR1/6 and TLR2, and TLR4, respectively. Ligand stimulation recruits MyD88 and TIRAP to TLRs, a complex of IRAKs and TRAF6 is subsequently formed, and results in NF‐κB activation. LPS induces TLR4 translocation to the endosome together with TRAM. TLR3 recognizes dsRNA in the endosomes. TLR3 and TLR4 activate TRIF‐dependent signalling, which activates NF‐kB and IRF3/7 resulting in the induction of proinflammatory cytokine genes and type I IFNs. In addition, 14‐3‐3θ can inhibit TLR2‐mediated activation of NF‐κB, but promote the activation of TLR4‐dependent transcription factors. 14‐3‐3ζ promotes the multimerization of TICAM‐1 (also known as TRIF) to form the TIC signalosome and regulate the TLR3‐TICAM‐1 signalling pathway

14‐3‐3 proteins are involved in viral infections, where they can be a key regulator for the expression of host and viral proteins.^65^ Toll‐like receptors (TLRs) are important pattern recognition receptors. 14‐3‐3 proteins are key regulators of TLR3 signalling and thus participate in innate immune regulation^66^ 14‐3‐3ζ participates in the TLR3–TICAM‐1 signalling pathway by promoting multimerization of TICAM‐1 (also known as TRIF) to form a TICAM‐1 signalosome.^66^ Myeloid differentiation primary response protein 88 (MyD88) is a common downstream adaptor recruited by all TLRs, with recruitment leading to activation of nuclear factor‐κB (NF‐κB).^67^ 14‐3‐3 proteins are also involved in the regulation of TLR4 signalling. On lipopolysaccharide (LPS) stimulation, PKCε is phosphorylated at Ser346 and Ser368, and is subsequently recruited to TLR4 in a MyD88‐dependent manner. The 14‐3‐3β isoform participates in the regulation of TLR4 by interacting with phosphorylated PKCε in a MyD88‐dependent manner.^68^ Differential effects on TLR2 and TLR4 signalling were observed for 14‐3‐3θ: it inhibited TLR2‐mediated NF‐κB activation but enhanced TLR4‐dependent transcription factor activation (Figure 2).^69^

### 5.2 14‐3‐3 proteins modulate antiviral defences via the RLR signalling pathway

Retinoic acid‐inducible gene I (RIG‐I)‐like receptors (RLRs) can initiate an immune response to RNA virus infection and are primarily sensed by RIG‐I and Melanoma differentiation‐associated gene 5 (MDA5). The 14‐3‐3ε isoform binds to RIG‐I and is an essential partner in the translocation complex with RIG‐I and the tripartite motif protein 25 (TRIM25) that guides the redistribution of RIG‐I from the cytosol to the membrane during an acute RNA virus infection for subsequent immune signalling.^70^, ^71^

The 14‐3‐3 proteins represent a molecular scaffold for stabilizing interactions between RIG‐I and TRIM25 proteins.^72^ Interaction between 14‐3‐3η and MDA5 can accelerate activation of MDA5 signalling, thereby helping host cells to mount a rapid and effective response against viral infections.^73^ The Zika virus NS3 protein physically interacts with 14‐3‐3ε and 14‐3‐3η, which hinders transfer of RIG‐I and MDA5 from the cytoplasm to mitochondria.^5^ Sendai virus (SeV), vesicular stomatitis virus (VSV), West Nile virus (WNV) and hepatitis C virus (HCV) are all sensed by both RIG‐I and MDA5.^74^ Both VSV and SeV can interact with RIG‐I receptors.^75^, ^76^ It has also been reported that 14‐3‐3ε forms a RIG‐I translocon with RIG‐I and TRIM25 following SeV or VSV infection to promote transfer of RIG‐I from the cytoplasm to the mitochondrial membrane.^71^ In the context of HCV infection, 14‐3‐3η promotes MDA5‐mediated activation of the antiviral signalling pathway.^72^ The WNV protein NS3 prevents RIG‐I binding to the receptor protein MAVS by interacting with 14‐3‐3ε, which blocks the RIG‐I‐mediated antiviral signalling pathway (Figure 2).^77^

14‐3‐3 proteins also play vital roles in DNA viruses through the RLR signalling pathway. The N‐terminal domains of herpesvirus large tegument proteins encode a conserved cysteine protease with ubiquitin‐ and NEDD8‐specific deconjugase activity (eg homologue BPLF1) that may regulate the RLR signalling pathway by interacting with 14‐3‐3. The EBV homologue BPLF1 interacts with 14‐3‐3 to promote the formation of a three‐molecule complex comprising 14‐3‐3, the ubiquitin ligase TRIM25, and RIG‐I, and deubiquitinates RIG‐I to resist the innate immune response.^6^ HCMV‐UL48 and KSHV‐ORF64 have the same function as BPLF1 in inhibiting the IFN response by targeting the 14‐3‐3–TRIM25 complex. HSV‐UL36 fails to induce TRIM25 autoubiquitination and aggregate formation because of a weaker interaction with 14‐3‐3.^7^ 14‐3‐3 proteins can affect viral infection (eg African swine fever virus by inhibiting cell apoptosis) and are considered to be a potential biomarker for HIV‐related neurodegeneration (Figure 2).^78^, ^79^

### 5.3 Other pathways

The ability to bind specific 14‐3‐3 proteins may allow viruses to both manipulate TLR and RLR signalling, and modulate other cellular processes. Members of the 14‐3‐3 protein family can regulate innate immunity and participate in the regulation of antiviral activity via many signalling pathways (eg MAPK, PI3K–AKT, NF‐κB and mTOR pathways).^80^, ^81^, ^82^ The IκB kinase (IKK) complex is a key regulator of the NF‐κB transcription factor, which directly controls two key steps for MEK‐1/2 kinase TPL‐2 activation in the inflammatory response. IKK complex phosphorylation of the TPL‐2 C‐terminus induces an association between 14‐3‐3 and tumour progression locus 2 (TPL‐2), stimulating TPL‐2 MEK‐1 kinase activity, which is essential for TPL‐2‐mediated activation of extracellular signal‐regulated kinase‐1/2 (ERK‐1/2). Binding of 14‐3‐3 to TPL‐2 is also indispensable for its induction of tumour necrosis factor alpha (TNF‐α), which is regulated independently of ERK‐1/2 activation.^77^ Moreover, 14‐3‐3 can activate the PI3K–AKT pathway in T cells to participate in the immune response.^83^ Protein kinase C (PKC) comprises a family of phospholipid‐dependent serine/threonine kinases that regulate diverse cellular functions and play an important role in immunity (eg autophagy).^62^ It has been reported that 14‐3‐3ε modulates PKCα activity.^84^ In addition, 14‐3‐3 proteins can regulate the mTOR pathway, connecting 14‐3‐3 with the autophagy regulatory processes involved in immunity.^29^ It has been reported that 14‐3‐3γ can regulate the mTOR signalling pathway to attenuate the inflammatory response induced by LPS in mammary epithelial cells in dairy cows and promote both cell proliferation and lactation.^85^ Furthermore, 14‐3‐3 controls the production of proinflammatory cytokines (eg IL‐6, IL‐8 and TNF‐α) via its participation in different signalling pathways in which it binds to signalling proteins with various functions, including kinases and transmembrane receptors.^65^ Related research revealed that human surfactant protein A (SP‐A) plays an important role in host defence, regulation of inflammation and surfactant metabolism in the lung.^86^ Isoforms of the 14‐3‐3 protein family can affect the different regulatory functions of SP‐A1 and SP‐A2 via direct binding to exon B of the SP‐A2 5′‐untranslated region.^87^SARS‐CoV‐2 infection can be aggravated by the imbalance of host innate immune response, leading to high incidence rate and lethality of COVID‐19.^88^ Type I interferon is an important effector molecule involved in antiviral immunity.^89^ The ORF6, ORF8 and N protein of SARS‐CoV‐2 were screened to inhibit the expression of IFN‐β, NF‐κB and IRSE promoter.^90^ These give hints of the potential role of 14‐3‐3 proteins in regulating the innate immune response caused by SARS‐CoV‐2, which provides a way of the treatment of COVID19. COVID‐19 is also related to neurological deficits mainly by interacting with 14‐3‐3 ζ and ε isoforms, which likely to become the target proteins of SARS‐CoV‐2 in the nervous system.^91^, ^92^

## 6 THERAPEUTIC TARGETING OF 14‐3‐3 PROTEINS

The literature contains strong evidence regarding the role played by seven human 14‐3‐3 isoforms in both cancer and neurodegenerative diseases.^93^ Protein–protein interactions (PPIs) has now been widely recognized as an attractive means to therapeutically intervene in disease states, in which the modulation of 14‐3‐3 PPIs plays important roles.^3^ Because the interaction of small molecules can regulate the inhibition and stabilization of 14‐3‐3 PPIs, it is a matter of time before novel pharmacological intervention is applied in clinical trials, including the therapeutic targeting to cancer, neurodegeneration, metabolic diseases, infection, and cystic fibrosis, and also in terms of drug discovery.^3^

Specific inhibitory peptides of 14‐3‐3 proteins may have potential in this context. For example, the 14‐3‐3 protein–target protein inhibitor R18 was identified in a phage display screen.^94^ Further studies revealed that R18 can inhibit all of the 14‐3‐3 protein family members with very similar affinity coefficients.^95^ There is a central sequence (WLDLE) in the amphipathic‐binding groove of 14‐3‐3 among the 14‐3‐3 complex crystal structure, which allows R18 to effectively utilized this amphiphilic property of 14‐3‐3 binding channel to compete for phosphorylated and non‐14‐3‐3‐dependent PPI.^96^ Besides R18, other peptide segments of 14‐3‐3 recognition sites can be used to design inhibitory peptides to intervene in the biological functions of 14‐3‐3 and associated disease processes. Difopein is an R18 dimer that can be used in mitigating viral infection, which binds to 14‐3‐3 proteins with high affinity, indicating that small molecule 14‐3‐3 modulators can be involved in regulating immune function or as antiviral agents.^97^

There are some other specific therapeutics identified and can also affect 14‐3‐3 PPI. For example, novel fragments have been reported recently to bind specifically to a lysine at the PPI interface of the p65‐subunit‐derived peptide of NF‐κB with the adapter protein 14‐3‐3.^98^ Other molecules have been discovered targeting p65/14‐3‐3. Dp‐005, a semi‐synthetic natural product derivative, binds and stabilizes the interface pocket of p65/14‐3‐3 complex.^99^ Besides, a conceptual molecule has been reported which is helpful to 14‐3‐3 stabilizer that interacts with glucose response element‐binding protein (ChREBP).^100^ There is fragments that represent promising starting points for the development of specific 14‐3‐3 PPI stabilizers has also been reported resently.^101^

Studies have shown that some small‐molecule anticancer agents can prevent binding of 14‐3‐3 by inhibiting phosphorylation of the target protein. For example, UCN‐01 can inhibit the activity of CHK1, TAK, CHK2 and other kinases, and thus phosphorylation of Ser216, the 14‐3‐3–binding site on CDC25C. This then prevents binding of 14‐3‐3 to CDC25C.^102^

## 7 CONCLUSIONS

New functions of 14‐3‐3 are increasingly being discovered. The 14‐3‐3 protein family plays a significant role in the cell cycle, apoptosis, autophagy, cell signal transduction, viral infections, innate immunity, disease and other unknown cellular signalling pathways. The multiple functions of 14‐3‐3 proteins make them a potential drug target for the treatment of disease or pathogen infection; furthermore, the modulation of 14‐3‐3 PPIs can help for discovering small molecular inhibitors and stabilizers, thereby we can therapeutically intervene in disease states. There are some specific therapeutics identified so far and can also affect the interaction of 14‐3‐3 with other targets. Further details regarding 14‐3‐3 ligands and functions remain to be discovered. We believe that identification of more specific biologics targeting 14‐3‐3 protein interactions will be helpful in the treatment of disease.

## CONFLICT OF INTEREST

We declare we have no competing interests.

## AUTHOR CONTRIBUTION

**Jiaqi Liu:** Writing‐original draft (lead). **Shengliang Cao:** Writing‐original draft (equal). **Guofei Ding:** Writing‐original draft (supporting). **Bin Wang:** Writing‐original draft (supporting). **Yingchao Li:** Writing‐original draft (supporting). **Yuzhong Zhao:** Writing‐original draft (supporting). **Qingyuan Shao:** Writing‐original draft (supporting). **Jian Feng:** Writing‐original draft (supporting). **Sidang Liu:** Writing‐review & editing (supporting). **Liting Qin:** Conceptualization (equal). **Yihong Xiao:** Conceptualization (lead); Writing‐review & editing (lead).

## Data Availability

This article has no additional data.
